# Development of a molecular maker for sex identification in Thai commercial date palm (*Phoenix dactylifera* L.)

**DOI:** 10.5511/plantbiotechnology.23.1214b

**Published:** 2024-03-25

**Authors:** Nuttapol Promkaew, Puangpaka Umpunjun, Ngarmnij Chuenboonngarm, Unchera Viboonjun

**Affiliations:** 1 Department of Plant Science, Faculty of Science, Mahidol University, Bangkok 10400, Thailand

**Keywords:** date palm, HAT-RAPD, SCAR marker, sex identification, sex-specific marker

## Abstract

Date palm (*Phoenix dactylifera* L.) is a dioecious plant, with male and female plants having distinct characteristics. Female plants are responsible for fruit production, and only approximately 10% of male plants are necessary for effective pollination. The determination of plant sex occurs during the first flowering, a process that typically spans 3–7 years. However, this extended timeframe results in significant time and valuable plantation resources being expended in the maintenance of trees. To address this issue, the study focused on sex identification of date palms using DNA markers. The research aimed to develop sex-specific markers for certain date palm cultivars, employing the high annealing temperature random amplified polymorphic DNA (HAT-RAPD) technique for accurate and reliable sex identification. In this investigation, 45 RAPD primers underwent screening in both male and female date palm plants to pinpoint sex-specific markers. Out of the total primers tested, only one, OPW-18, exhibited a correlation with sex. OPW-18 produced a distinct band of approximately 400 bp, consistently present in all male plants but absent in all female plants. The male-specific fragment from OPW-18 was cloned and sequenced to facilitate the development of sex-specific sequence-characterized amplified region (SCAR) primers. The outcomes revealed that the newly crafted SCAR primer pair, mspW18-2F and mspW18-2R, successfully amplified a unique fragment of 283 bp exclusively in male plants. This capability allowed the identification of 100% of male plants in the KL1 and Barhi cultivars. These markers prove to be efficient, reliable, and reproducible for early-stage sex identification in plants.

## Introduction

Date palm (*Phoenix dactylifera* L.) is a dioecious species, featuring distinct male and female individuals with unisexual flowers. Male and female plants exhibit varying characteristics. Date palms contribute significantly to various products beneficial to humans, with the primary product being the date fruit. This versatile fruit can be consumed fresh, dried, or in various processed forms. Notably, dates offer essential benefits as they are rich in energy, crucial nutrients, and fiber. Mature dates are considered high-energy foods, containing sugar levels ranging from 72% to 88%. Additionally, date fruits serve as excellent sources of iron and potassium. Moreover, dates provide amino acids and vitamin A (Chao and Krueger 2007).

Like other dioecious plants, female date palm plants are responsible for fruit production, while male plants serve solely for pollination purposes. Approximately 10% of male plants are necessary for effective pollination in the cultivation of date palms (Dhawan et al. 2013). Given that the determination of sexuality is not feasible before floral initiation, the development of methods for sex identification in dioecious plants during the juvenile stage would significantly benefit breeding programs focused on fruits and seeds. Early identification and removal of excess male plants can prevent the unnecessary depletion of vital plantation resources. Various techniques, including chromosome identification and molecular markers, have been devised for distinguishing between male and female plants.

The sex ratio of date palm progeny, as reported by Saadi in 1990, indicates a 1 : 1 distribution, implying genetic sex determination through a single locus. Subsequent research proposed an XY chromosome system, drawing on observations of heteromorphic chromocenters in male interphase nuclei, suggesting its role as a sex determinant in date palms (Siljak-Yakovlev et al. 1996). Chromosome analysis often requires skilled personnel and specialized laboratory equipment. The process of sample collection, preparation, and analysis can be time-consuming, especially for large populations. In a recent development, sex-linked DNA markers were detected in date palm through a PCR-based method (Intha and Chaiprasart 2018). This advancement allows for the identification of plants at the early seedling stage, resulting in significant time, space, and cost savings compared to the conventional practice of growing trees in traditional plantations until they reach the reproductive stage. Nevertheless, there is a need to broaden the pool of available sex-linked DNA markers to encompass all cultivars planted not only globally but specifically in Thailand.

Various molecular genetic techniques have been explored for sex identification in different plant species. Among these, randomly amplified polymorphic DNA (RAPD) stands out as one of the most widely employed methods, utilizing a DNA fingerprinting approach based on polymerase chain reaction (PCR). RAPD offers a straightforward and cost-effective solution, requiring no prior specific knowledge about the target plant or primer design.

This study specifically opted for the high annealing temperature random amplified polymorphic DNA (HAT-RAPD) technique, as introduced by Anuntalabhochai et al. (2000). This technique, an advancement of the RAPD method, boasts high accuracy and reproducibility (Anuntalabhochai et al. 2000; Atienzar et al. 2000; Chundet et al. 2007; Meesangiem et al. 2017; Siritheptawee et al. 2018; Sripalwit et al. 2007; Suttaduk et al. 2015). Its reliance on elevated temperatures enhances primer stringency during binding to single-stranded template DNA, particularly in regions with complementary bases. Notably, the procedural steps are uncomplicated, and the overall cost is relatively low compared to alternative molecular marker techniques.

The enhancement of PCR assays for molecular identification is achieved through the conversion of RAPD fragments and other molecular markers into SCAR (Sequence Characterized Amplified Region). This process involves sequencing specific DNA bands and designing locus-specific primers, thereby improving reproducibility and reliability (Paran and Michelmore 1993). In this current study, we employed molecular techniques to develop sex-specific SCAR markers for select date palm cultivars, aiming to ensure dependable sex identification in date palm plants.

## Materials and methods

### Plant material

The plant materials investigated in this study consist of sexually mature date palm trees obtained from seedlings of two commercially significant cultivars in Thailand: the KL1 and Barhi cultivars. We determined their sexual identification as male or female through multiple flowering occurrences. To identify and develop sex-specific markers, we collected young leaves from fully mature trees, representing a total of 62 individuals (Supplementary Table S1). The samples comprised 31 male and 31 female individuals, originating from both KL1 and Barhi. The trees were located across three horticulture farms in different regions: 1) the Horticulture Farm in Pak Tho District, Ratchaburi Province; 2) the Horticulture Farm in Minburi District, Bangkok; and 3) the Horticulture Farm in Therdthai Subdistrict, Bang Khae District, Bangkok. Notably, the KL1 date palm is a hybrid between Deglet Nour and Barhi cultivars, using pollen of the Deglet Nour (a cultivar commonly eaten dried) from Israel and female flowers of the Barhi (a popular cultivar for eating fresh fruit) from Jordan.

### Genomic DNA isolation

Genomic DNA extraction involved utilizing the UniversAll™ Tissue Extraction Kit from Yeastern Biotech Inc., Taiwan, specifically employing UniversAll™ Extraction Buffer for isolation from young leaf samples. Each 2 mm×2 mm leaf sample was treated with 52 µl of a mixed solution containing 50 µl of Extraction Buffer and 2 µl of Extraction Enhancer in a microcentrifuge tube, ensuring complete submersion in the buffer. Subsequently, the tube underwent incubation at 98°C for 10 min, followed by vortexing and brief centrifugation. Post-extraction, all DNA samples were quantified using a Nanodrop® ND-1000 UV-Vis Spectrophotometer, diluted to a concentration of 50 ng µl^−1^, and stored at −20°C.

A total of 45 RAPD primers (Supplementary Table S2) from Operon Technologies Inc., USA, were employed to amplify DNA samples, focusing on achieving clear and reproducible amplification banding patterns. Each reaction for RAPD-PCR comprised 1x PCR buffer, 100 µM dNTPs, 1 µM primer, 1.5 mM MgCl_2_, 1 U Taq DNA polymerase, and 50 ng of template DNA. The PCR amplifications followed a protocol: an initial denaturation step of 4 min at 94°C, followed by 40 three-step amplification cycles. Each cycle consisted of 1 min at 94°C, 1 min at the annealing temperature (50°C), and a 2-min extension at 72°C. A final extension step at 72°C for 5 min was included. Subsequently, all PCR-amplified DNA fragments were separated by electrophoresis in submerged horizontal agarose gels (1.5% w/v), stained with Midori Green Advanced DNA Stain (Nippon Genetics Inc., Japan), and visualized under ultraviolet (UV) light.

### Cloning and sequencing of sex-specific RAPD marker

The sex-specific fragment, amplified by the RAPD marker, was extracted from the agarose gel using the FavorPrep GEL/PCR Purification Mini Kit from Favogen Biotech Corp., USA. Subsequently, it was inserted into the pGEM-T easy vector from Promega Corp., USA. The DNA fragment within the vector was then amplified using universal primers, and the resulting product was subjected to sequencing through the Barcode-Tagged Sequencing method by Celemics Inc., Republic of Korea.

### Conversion of RAPD marker into sex-specific SCAR primers and PCR amplification

Sex-specific primer pairs were designed utilizing the Primer3Plus tool (https://www.bioinformatics.nl/cgi-bin/primer3plus/primer3plus.cgi) based on the sequence of sex-specific RAPD markers. These primer pairs were then evaluated on the DNA of male and female date palms through PCR amplification. Each PCR reaction comprised 1x PCR buffer, 100 µM dNTPs, 1 µM primer, 1.5 mM MgCl_2_, 1 U Taq DNA polymerase, and 50 ng of template DNA. To avoid false-positive results, a negative control was performed using water instead of a DNA template. The PCR amplifications followed a procedure: an initial denaturation step of 5 min at 94°C, succeeded by 30 three-step amplification cycles. Each cycle consisted of 30 s at 94°C, 30 s at the annealing temperature (52.5°C), and 30 s of extension at 72°C. A final extension step at 72°C for 7 min was included. Subsequently, all PCR-amplified DNA fragments were separated by electrophoresis in submerged horizontal agarose gels (1.5% w/v), stained with Midori Green Advanced DNA Stain, and visualized under ultraviolet (UV) light.

## Results

### Genomic DNA isolation and HAT-RAPD analysis

**Figure 1:**
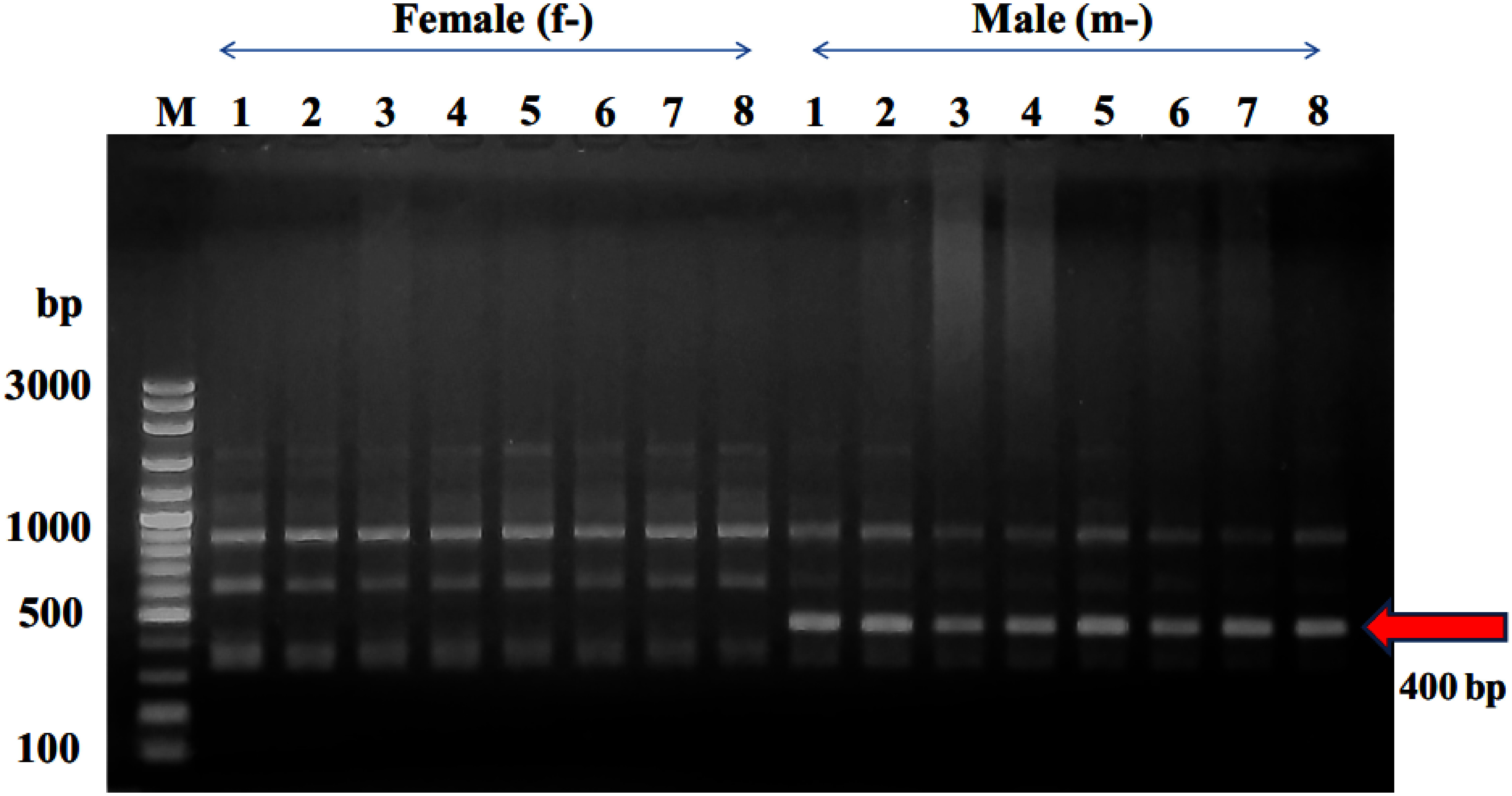
Figure 1. RAPD profiles depicting male and female KL1 date palm individuals, generated by primer OPW-18. The arrow highlights the male-specific fragment of approximately 400 bp. In the gel lanes, f-1 to f-8 represent female individuals, while m-1 to m-8 represent male individuals. The letter “M” designates the 100-bp DNA ladder.

The genomic DNA successfully extracted from the leaf using the UniversAll™ Tissue Extraction Kit. Subsequently, the HAT-RAPD method, employing 45 random primers, was utilized to analyze the DNA polymorphism. The findings revealed that the OPW-18 primer uniquely highlighted distinctions in DNA polymorphism between male and female date palms within the KL1 cultivar. Specifically, a distinctive band at approximately 400 bp was observed in the DNA polymorphism of male date palms (Figure 1).

### Cloning and sequencing of sex-specific RAPD marker

**Figure 2:**
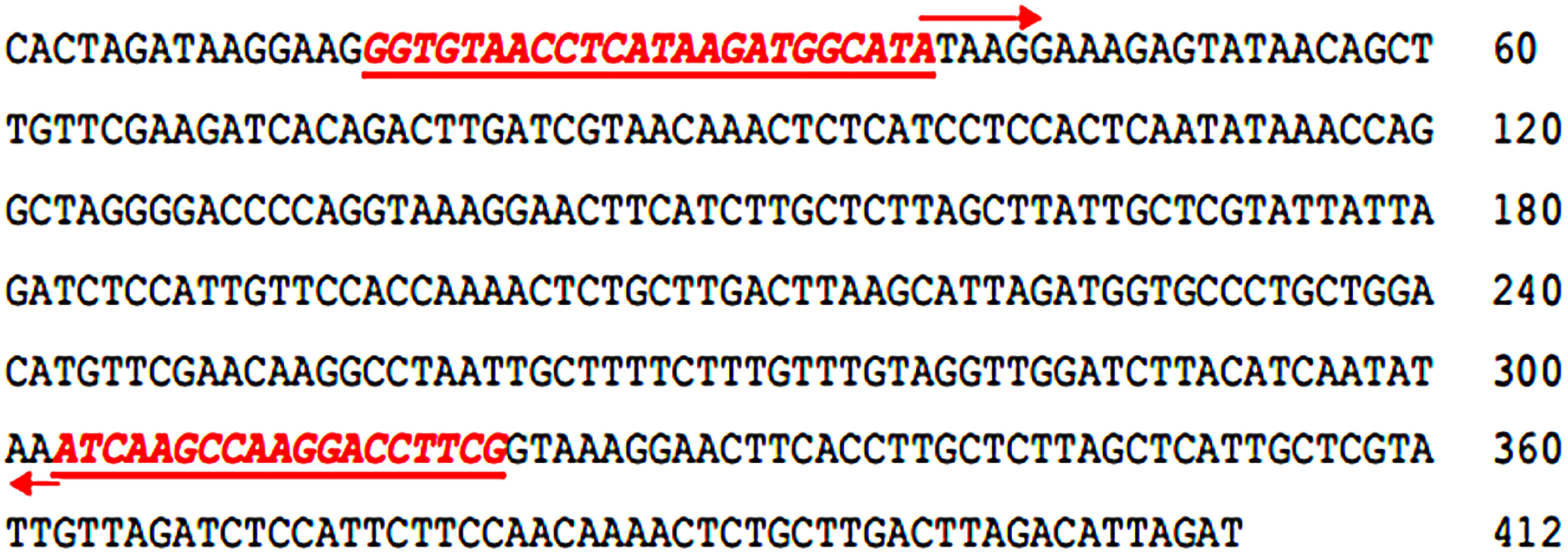
Figure 2. The nucleotide sequence (412 bp) of the cloned male-specific RAPD fragment. Boldfaced and italicized nucleotides, highlighted, denote the forward mspW18-1F and reverse mspW18-1R SCAR primers.

**Figure 3:**
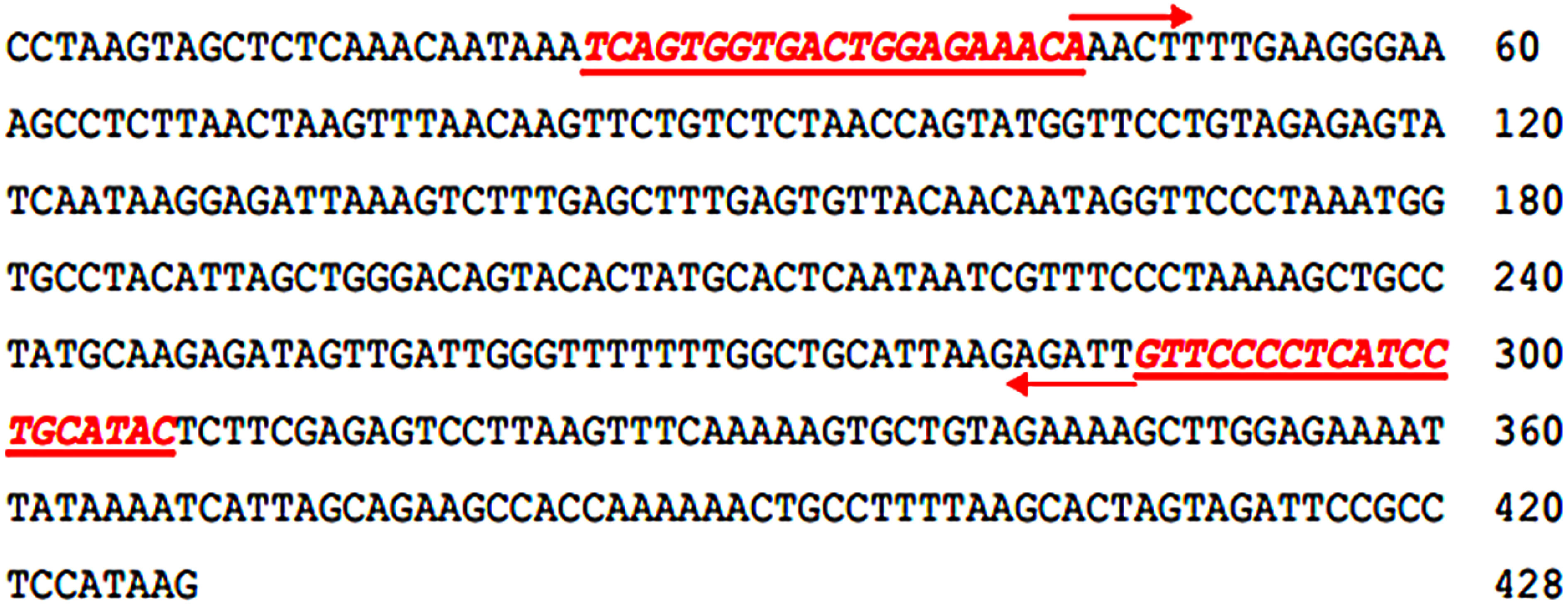
Figure 3. The nucleotide sequence (428 bp) of the cloned male-specific RAPD fragment. Boldfaced and italicized nucleotides, highlighted, denote the forward mspW18-2F and reverse mspW18-2R SCAR primers.

The male-specific fragment of approximately 400 bp, amplified by the OPW-18 primer, underwent cloning, sequencing, and subsequent comparison with data available in GenBank. After sequencing, the male-specific fragments revealed two different sequences of 412 bp and 428 bp, respectively (Figures 2, 3). Additionally, these two sequences share only 24.03% homology as shown in Supplementary Figure S1. These sequences exhibited no homology with any sequence in the database upon conducting a BLASTn search (http://www.ncbi.nlm.nih.gov/Blast.cgi). Accordingly, these DNA regions might not be directly related to the locus controlling sex.

### Conversion of RAPD marker into sex-specific SCAR primers and PCR amplification

**Figure 4:**
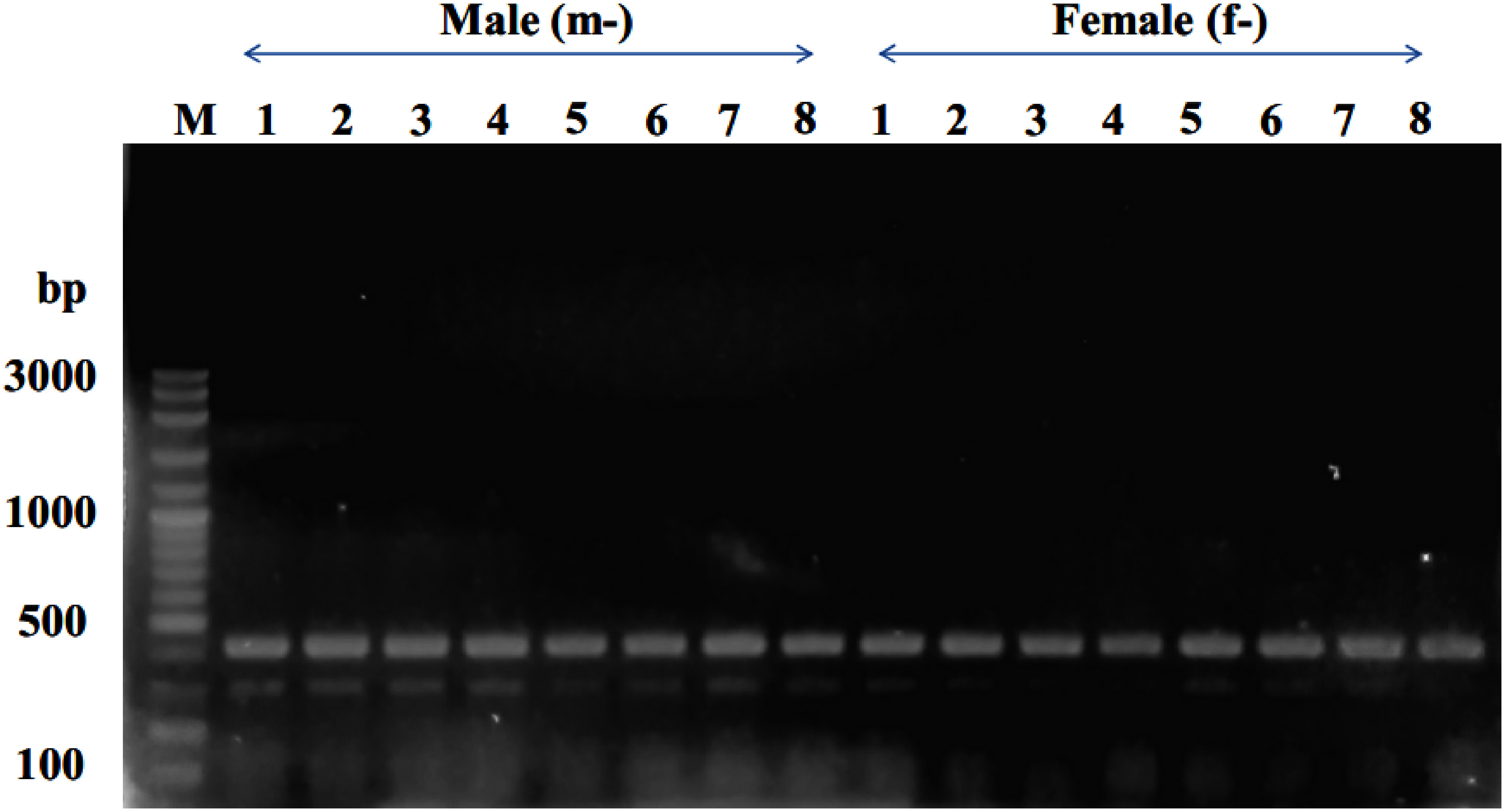
Figure 4. Amplicon profiles of male and female KL1 date palm individuals, illustrating the products generated by the mspW18-1F/mspW18-1R SCAR primers. In the gel lanes, m-1 to m-8 represent male individuals, while f-1 to f-8 represent female individuals. The letter “M” designates the 100-bp DNA ladder.

**Figure 5:**
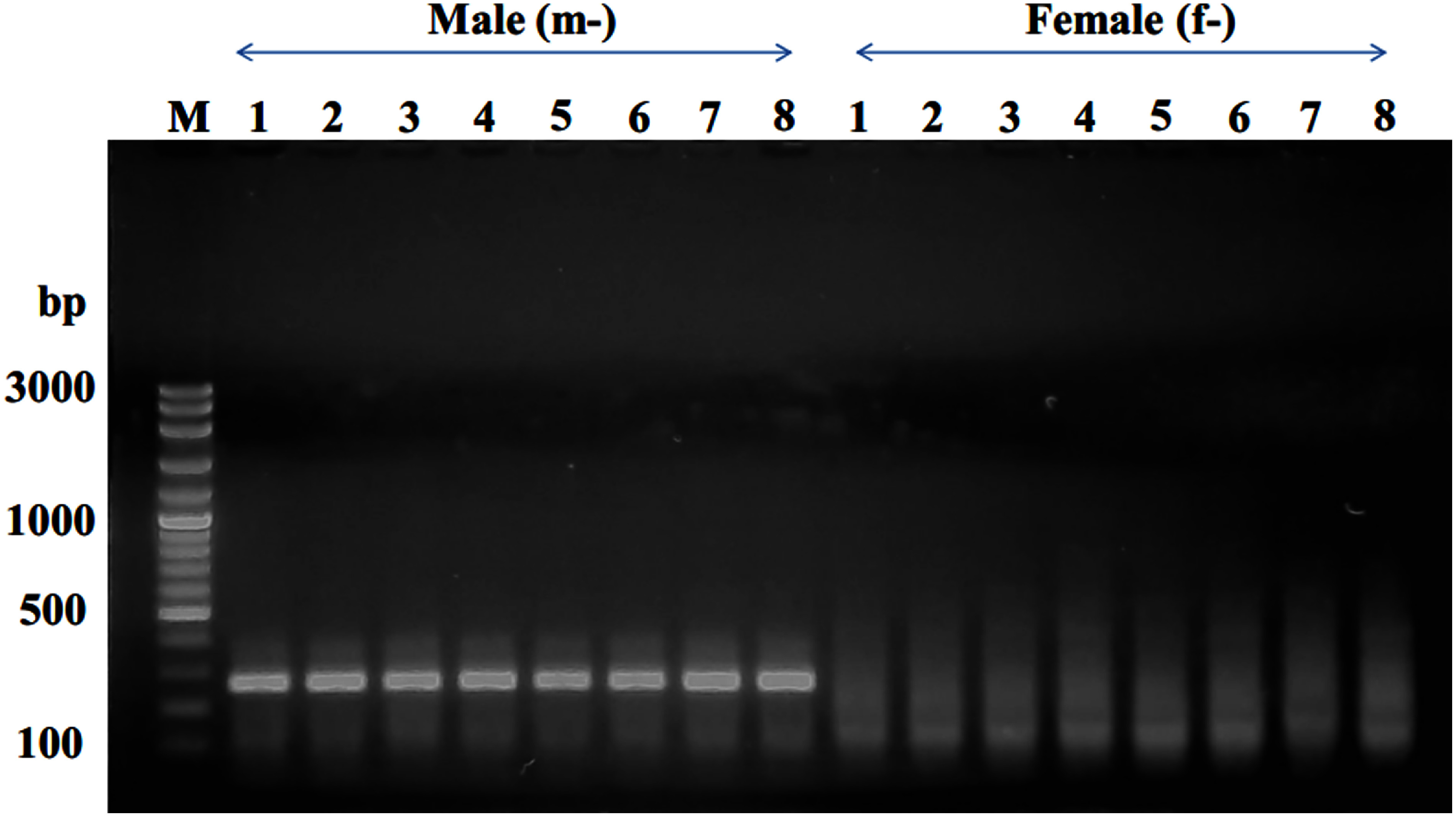
Figure 5. Amplicon profiles of male and female KL1 date palm individuals, illustrating the products generated by the mspW18-2F/mspW18-2R SCAR primers. In the gel lanes, m-1 to m-8 represent male individuals, while f-1 to f-8 represent female individuals. The letter “M” designates the 100-bp DNA ladder.

Sex-specific SCAR primer pairs were designed based on the sequence of sex specific RAPD fragments with Primer3Plus as shown in Figures 2–3 and Table 1. The SCAR primer pair (mspW18-1F/mspW18-1R) was applied to 16 individual KL1 date palm samples (comprising 8 males and 8 females). However, the resulting amplicons of approximately 300 bp and 400 bp were observed in both male and female samples (Figure 4). This outcome indicates that this specific primer pair cannot be utilized to reliably identify the sex of KL1 cultivar. Alternatively, the SCAR primer pair (mspW18-2F/mspW18-2R) underwent testing on 16 individual KL1 date palm samples. The result revealed the presence of a unique amplicon of 283 bp exclusively in male samples (Figure 5). This establishes it as a novel male-specific SCAR marker, demonstrating its utility in identifying the sex of the KL1 date palm cultivar.

**Table 1:** Table 1. SCAR primers developed in this study.

SCAR primer name	Primer sequence
mspW18-1F	5′ GGTGTAACCTCATAAGATGGCATA 3′(24 base)
mspW18-1R	5′ CGAAGGTCCTTGGCTTGAT 3′(19 base)
mspW18-2F	5′ TCAGTGGTGACTGGAGAAACA 3′(21 base)
mspW18-2R	5′ TTGGTGGCTTCTGCTAATGA 3′(20 base)

**Figure 6:**
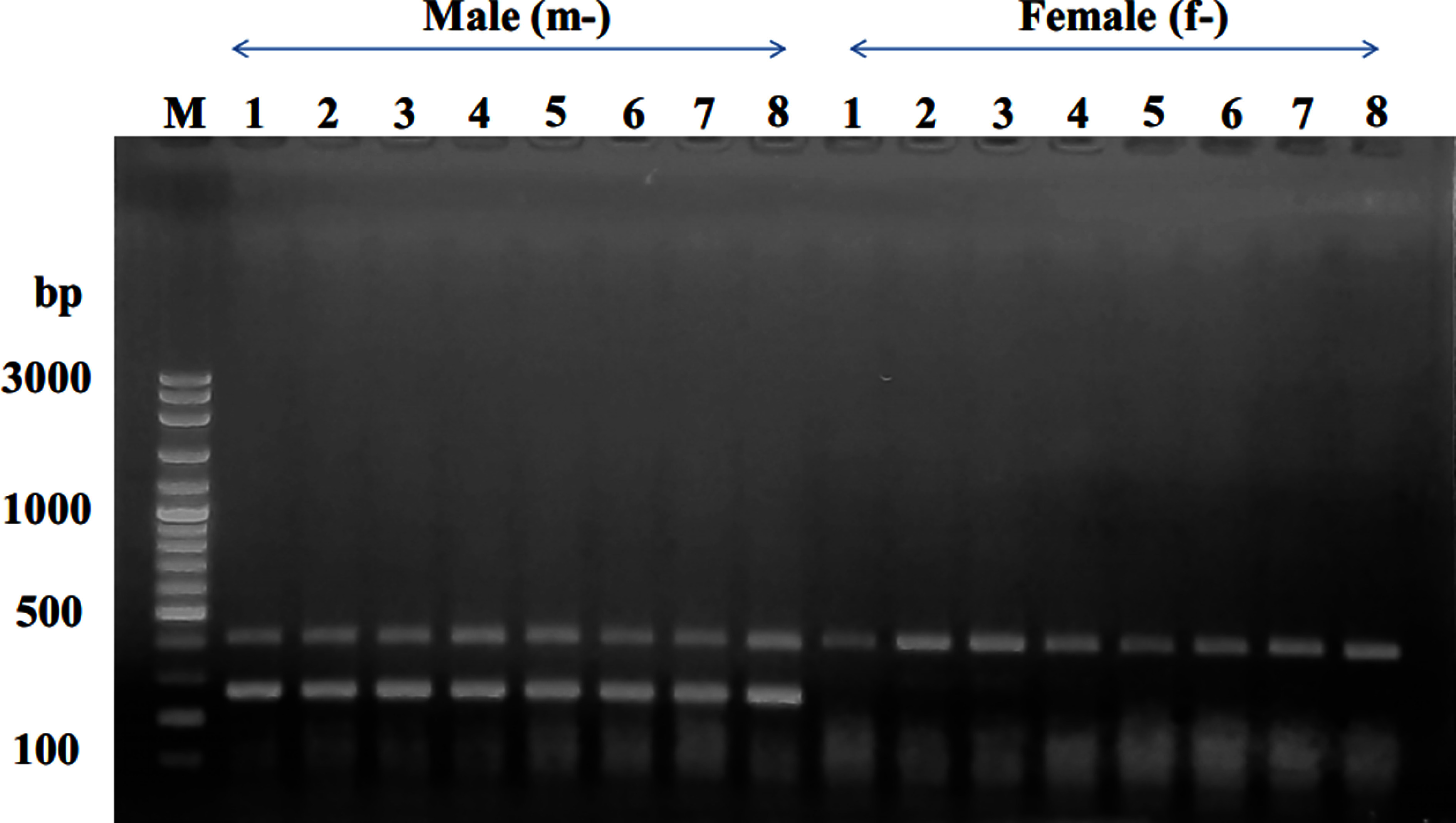
Figure 6. Amplicon profiles of male and female KL1 date palm individuals, illustrating the products generated by a multiplex PCR reaction utilizing the mspW18-2F/mspW18-2R primer pair in combination with the mspW18-1F/mspW18-1R primer pair. In the gel lanes, m-1 to m-8 represent male individuals, while f-1 to f-8 represent female individuals. The letter “M” designates the 100-bp DNA ladder.

In a study involving 16 samples of KL1 date palm, the multiplex PCR reaction incorporated the mspW18-2F/mspW18-2R primer pair along with the mspW18-1F/mspW18-1R primer pair. The investigation revealed that the mspW18-1F/mspW18-1R primer pair augmented the presence of approximately 400-bp DNA fragments in both male and female plants. Conversely, the mspW18-2F/mspW18-2R primer pair exclusively increased the count of 283 bp DNA fragments in male plants, manifesting as two distinctive DNA bands solely in male plants (Figure 6). This finding implies that the concurrent use of these primer pairs in a multiplex PCR reaction is proficient in discerning the sex of date palm trees, establishing it as a valuable marker for selecting both male and female plants.

**Figure 7:**
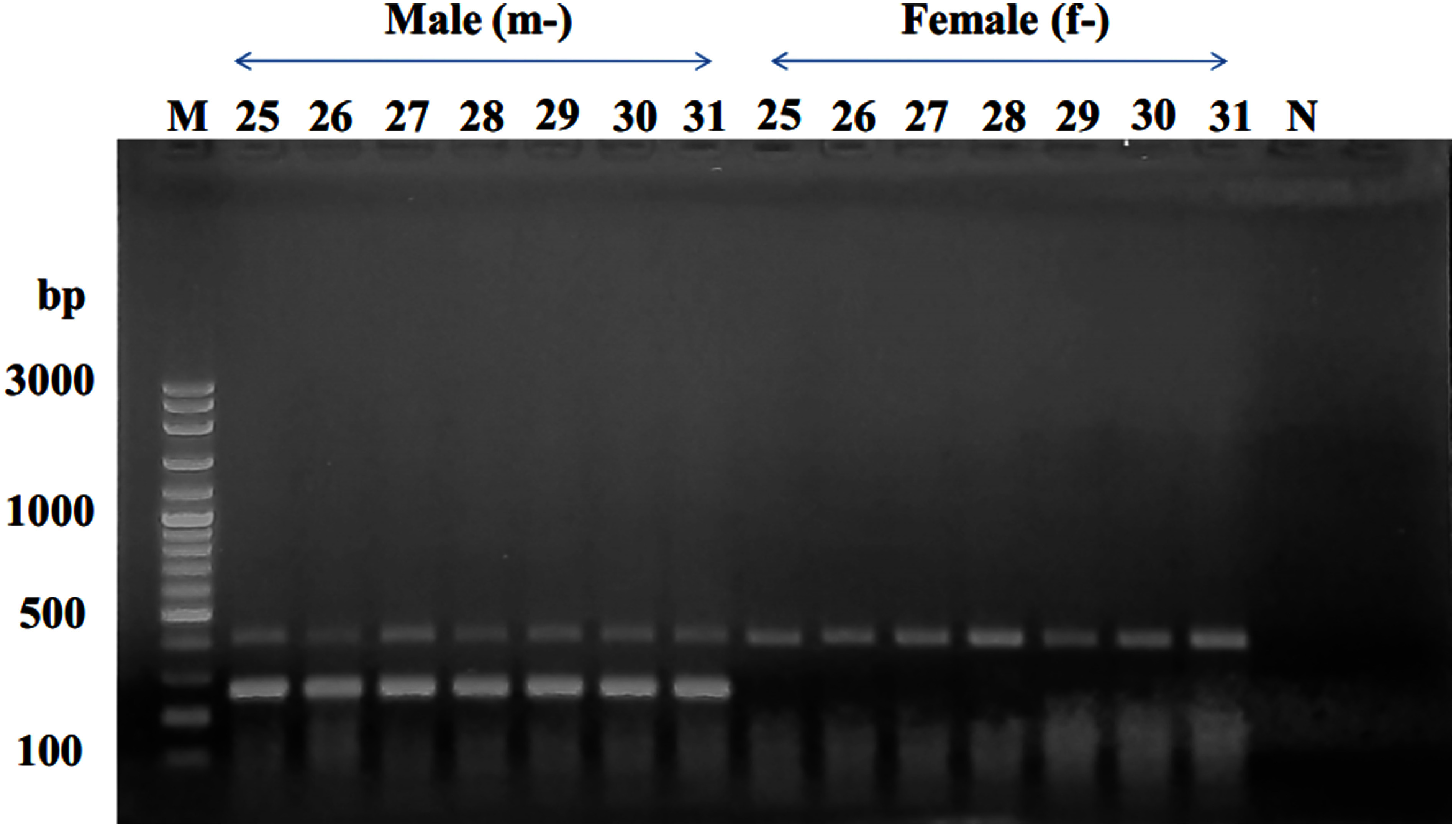
Figure 7. Amplicon profiles of male and female Barhi date palm individuals, illustrating the products generated by a multiplex PCR reaction utilizing the mspW18-2F/mspW18-2R primer pair in combination with the mspW18-1F/mspW18-1R primer pair. In the gel lanes, m-25 to m-31 represent male individuals, while f-25 to f-31 represent female individuals. The letter “M” designates the 100-bp DNA ladder. The letter “N” designates the negative control.

Subsequently, these two primer pairs were employed for testing in the mature seedling progenies from the Barhi cultivar. The results demonstrated that two DNA bands were evident in male samples, while only one DNA band appeared in female samples, mirroring the observations in KL1 date palm (Figure 7).

Upon additional examination of primer specificity and efficiency for determining date palm sex, a multiplex PCR reaction was conducted utilizing the mspW18-2F/mspW18-2R primer pair in combination with the mspW18-1F/mspW18-1R primer pair. This analysis involved 62 samples of KL1 and Barhi date palm with known sexes. The results revealed that the simultaneous utilization of these two primer pairs in the reaction achieved a 100% accuracy in identifying date palm sex (Supplementary Figure S2).

## Discussion

To cultivate date palms successfully, it is essential to have one male plant for pollination per every 9–10 female plants (Dhawan et al. 2013). Consequently, if farmers can determine the gender of date palm trees during the seedling or juvenile stage, it will enable them to strategize planting more efficiently, leading to time and cost savings. In cases where the sex is initially unknown, farmers must plant a date palm tree and wait 3–7 years to ascertain its gender, resulting in considerable time and expense devoted to maintaining the trees.

Previous studies, it has been found that date palms are plants with an XY chromosome system (Cherif et al. 2013; Siljak-Yakovlev et al. 1996). Therefore, sex can be identified using sex chromosomes at seedling stage by preparing chromosomes from root tips. However, in dioecious plants, male and female plants typically exhibit rare differences in sex chromosomes, and the existing methodologies for investigating plant sex chromosomes pose challenges for researchers. Consequently, molecular genetic approaches have been explored to distinguish the sex of diverse plant species (Biffi et al. 1995; Ehsanpour and Arab 2009; Gao et al. 2007; Reamon and Jung 2000; Sakamoto et al. 1995), including date palms. Among these techniques, randomly amplified polymorphic DNA (RAPD) has emerged as a popular choice—a cost-effective and straightforward DNA fingerprinting method based on polymerase chain reaction (PCR) (George et al. 2007; Hosseini et al. 2011; Ii et al. 2012; Niroshini et al. 2000; Panda et al. 2010; Samantaray et al. 2010, 2012; Shirkot et al. 2002). In this investigation, we opted for HAT-RAPD, a technique derived from the random amplified polymorphic DNA method, known for its heightened accuracy and reproducibility, ensuring consistent results upon repetition (Anuntalabhochai et al. 2000; Atienzar et al. 2000). The findings indicated that the HAT-RAPD technique, employing a primer annealing temperature of 50°C, generated numerous DNA bands with superior resolution compared to those produced by the RAPD technique using a primer annealing temperature of 37°C. The RAPD-derived DNA bands exhibited a smeared pattern, preventing clear individual band identification. These outcomes align with the findings of Anuntalabhochai et al. (2000), who reported that utilizing a primer annealing temperature within the range of 46 to 62°C results in the synthesis of multiple DNA bands with heightened clarity, facilitating reproducibility in experiments.

While sex-related RAPD and SCAR markers have been reported in date palms previously (Dhawan et al. 2013; Younis et al. 2008), their applicability to samples from unrelated cultivars remains unverified. Molecular marker analysis for gene-related markers typically involves screening a large number of primers, a necessity influenced by genome size and marker coverage. Younis et al. (2008) screened 30 primers, identifying 3 female-specific and 2 male-specific markers. Dhawan et al. (2013) tested 100 primers, obtaining only one male-specific marker. Al-Qurainy et al. (2018) tested 300 primers, with only one producing a reproducible band in male plants. Kethirun et al. (2023) utilized a total of 160 random decamer primers to screen for specific RAPD markers in off-season flowering male and female date palm populations. They identified only one marker that generated distinct genomic DNA patterns in female off-season flowering trees. In this investigation, a total of 45 RAPD primers were utilized with the objective of discovering a male-specific marker. The sequence of this particular marker exhibited no resemblance to any known sequences documented in the NCBI database. The primary significance of our research resides in the establishment of a novel SCAR marker for identifying the sex of date palms, based on the RAPD marker. Despite the effective differentiation between males and females achieved by the RAPD marker (OPW-18) using the HAT-RAPD technique, the SCAR marker offers distinct advantages over RAPD markers, particularly in terms of the ease of detecting specific sites on agarose gel.

Assessing the specificity of the newly devised primers for date palm sex through multiplex PCR, we employed dual primers mspW18-2F/mspW18-2R and mspW18-1F/mspW18-1R in conjunction with 62 known sex samples from KL1 and Barhi date palms (Supplementary Figure S2). The results revealed that when these two primer pairs were combined in the reaction, sex could be accurately distinguished with a 100% success rate. Notably, the primer pair mspW18-1F/mspW18-1R amplified DNA fragments of approximately 400 bp in both male and female plants, while the primer pair mspW18-2F/mspW18-2R selectively increased the DNA fragment of male trees by 283 bp, manifesting as two distinct DNA bands. Consequently, these primers can serve as a reliable marker for discriminating between male and female date palm trees. The outcomes of this study have demonstrated their efficacy as a superior option for sex identification, particularly in experiments where PCR product absence is encountered in the sample. This absence may be attributed to the lack of DNA or potential impediments in the PCR process, resulting in false negative outcomes (Milewicz and Sawicki 2013). Consequently, it is advisable to employ two distinct sex markers capable of amplifying male and female products of varying lengths within a single reaction. This approach helps address the challenges associated with PCR product absence, thereby enhancing the accuracy and reliability of sex identification.

The male-specific SCAR primers, mspW18-2F and mspW18-2R, derived from the RAPD sequence of the KL1 cultivar, underwent testing on the Barhi date palm. The outcome revealed that this primer pair yielded a distinctive band of 283 bp exclusively in males, mirroring the results observed in the KL1 date palm. However, when the same male-specific SCAR primers were applied to Nawader and Khonaizi date palms, the primers could not give any PCR products on these mature seedling progenies (unpublished data). The result implied that this primer pair might be specific to a particular cultivar. Consequently, these primers proved ineffective in determining the sex of Nawader and Khonaizi date palms. This discrepancy in primer effectiveness between Nawader and Khonaizi date palms compared to KL1 and Barhi may stem from the genetic similarity between KL1 and Barhi. The KL1 date palm is a cultivated variety developed through crossbreeding the Deglet Nour cultivar from Israel with the Barhi cultivar from Jordan. The process involved planting and selecting first-generation hybrid seeds until achieving a high-yielding cultivar (Intha and Chaiprasart 2018). The shared genetic background between KL1 and Barhi could explain the consistent results observed in these cultivars. However, the efficacy of sex identification in other date palm cultivars warrants further investigation in future studies.

In the technique developed by Intha and Chaiprasart (2018) using PCR, specific tetraprimers linked to the sex chromosome were employed. In male flowering date palms, two amplicons (430 bp and 320 bp) were observed, while female date palms displayed a single amplicon (430 bp). This method has proven to be effective in sex identification for various cultivars, including KL1, Deglet Nour, Barhi, Hayani, Medjool, and Tunisia, all of which consistently exhibited DNA banding patterns. Thus, primers designed based on sex chromosome sequences could be valuable for identifying the sex of individual plants across a variety of cultivars. It is noteworthy that these primers have not been utilized for Nawader and Khonaizi, which are other commercial cultivars in Thailand. Expanding the repertoire of sex determination markers for date palm cultivars, not only in Thailand but globally, could indeed be beneficial. Having a broader set of markers can enhance the accuracy and reliability of sex identification across diverse cultivars. This is particularly important as different cultivars may exhibit genetic variations that may not be captured by a single set of markers.

In conclusion, the HAT-RAPD technique was employed to screen RAPD primers in this study, yielding consistent outcomes for sex discrimination in date palm plants. The distinct band generated from male plants was sequenced, leading to the design of more targeted SCAR primers. These primers offer a practical tool for early-stage sex identification in date palm trees. The utilization of sex-specific DNA markers can assist farmers in managing the sex ratio of date palm plantations, preventing the excessive planting of male date palms, and saving both time and costs. These discoveries play a crucial role in tackling diverse challenges, notably in advancing the creation of DNA probes for identifying the sex of date palm trees through dot blot hybridization. Additionally, dot blot hybridization stands out as an expedient technique for swiftly determining the presence or absence of specific sequences.

## Data Availability

The other data that support this study are available in the article and accompanying supplementary material.

## Abbreviations

HAT-RAPD: high annealing temperature random amplified polymorphic DNA
PCR: polymerase chain reaction
SCAR: sequence-characterized amplified region
