# Supplementary material for: Development of a molecular maker for sex identification in Thai commercial date palm (Phoenix dactylifera L.)
Source: Plant Biotechnol (Tokyo). 2024 Mar 25;41(1):45–51. doi: 10.5511/plantbiotechnology.23.1214b (PMC11500566; doi:10.5511/plantbiotechnology.23.1214b)
Supplement: Supplementary Data [file plantbiotechnology-41-1-23.1214b-s001.pdf]

Supplementary Data

Table S1: Details of date palm trees (collected from Horticulture Farm Areas), used for identification and development of sex specific markers

No.	Code	Cultivar	Symbol	Sex	Location
1	f-1	KL1	KLFBK 1906-1	Female	Minburi District, Bangkok
2	f-2	KL1	KLFBK 1906-2	Female	Minburi District, Bangkok
3	f-3	KL1	KLFBK 1906-3	Female	Minburi District, Bangkok
4	f-4	KL1	KLFBK 1906-4	Female	Minburi District, Bangkok
5	f-5	KL1	KLFBK 1906-5	Female	Minburi District, Bangkok
6	f-6	KL1	KLFBK 1906-13	Female	Minburi District, Bangkok
7	f-7	KL1	KLFBK 1906-14	Female	Minburi District, Bangkok
8	f-8	KL1	KLFBK 1906-15	Female	Minburi District, Bangkok
9	f-9	KL1	KLFBK1906-16	Female	Minburi District, Bangkok
10	f-10	KL1	KLFBK 1906-17	Female	Minburi District, Bangkok
11	f-11	KL1	KLFBK 1906-18	Female	Minburi District, Bangkok
12	f-12	KL1	KLFBK 1906-19	Female	Minburi District, Bangkok
13	f-13	KL1	KLFBK 0711-10	Female	Minburi District, Bangkok
14	f-14	KL1	KLFBK 0711-11	Female	Minburi District, Bangkok
15	f-15	KL1	KLFBK 0711-12	Female	Minburi District, Bangkok
16	f-16	KL1	KLFBK 0711-13	Female	Minburi District, Bangkok
17	f-17	KL1	KLFBK 0711-14	Female	Minburi District, Bangkok
18	f-18	KL1	KLFBK 0711-15	Female	Minburi District, Bangkok
19	f-19	KL1	KLFBK 0711-16	Female	Minburi District, Bangkok
20	f-20	KL1	KLFBK 0711-17	Female	Minburi District, Bangkok
21	f-21	KL1	KLFRB 0603-6	Female	Pak Tho District, Ratchaburi
22	f-22	KL1	KLFRB 0606-7	Female	Pak Tho District, Ratchaburi
23	f-23	KL1	KLFRB 0603-8	Female	Pak Tho District, Ratchaburi

Table S1: Details of date palm trees (collected from Horticulture Farm Areas), used for identification and development of sex specific markers (cont.)

No.	Code	Cultivar	Symbol	Sex	Location
24	f-24	KL1	KLFRB 0603-9	Female	Pak Tho District, Ratchaburi
25	f-25	Barhi	BHFBK 1910-7	Female	Bang Khae District, Bangkok
26	f-26	Barhi	BHFBK 1910-8	Female	Bang Khae District, Bangkok
27	f-27	Barhi	BHFBK 2701-27	Female	Bang Khae District, Bangkok
28	f-28	Barhi	BHFBK 2701-28	Female	Bang Khae District, Bangkok
29	f-29	Barhi	BHFBK 2701-29	Female	Bang Khae District, Bangkok
30	f-30	Barhi	BHFBK 2701-30	Female	Bang Khae District, Bangkok
31	f-31	Barhi	BHFBK 2701-31	Female	Bang Khae District, Bangkok
32	m-1	KL1	KLMBK 1906-6	Male	Minburi District, Bangkok
33	m-2	KL1	KLMBK 1906-7	Male	Minburi District, Bangkok
34	m-3	KL1	KLMBK 1906-8	Male	Minburi District, Bangkok
35	m-4	KL1	KLMBK 1906-9	Male	Minburi District, Bangkok
36	m-5	KL1	KLMBK 1906-10	Male	Minburi District, Bangkok
37	m-6	KL1	KLMBK 1906-11	Male	Minburi District, Bangkok
38	m-7	KL1	KLMBK 1906-12	Male	Minburi District, Bangkok
39	m-8	KL1	KLMBK 0711-1	Male	Minburi District, Bangkok
40	m-9	KL1	KLMBK 0711-2	Male	Minburi District, Bangkok
41	m-10	KL1	KLMBK 0711-3	Male	Minburi District, Bangkok
42	m-11	KL1	KLMBK 0711-4	Male	Minburi District, Bangkok
43	m-12	KL1	KLMBK 0711-5	Male	Minburi District, Bangkok
44	m-13	KL1	KLMBK 0711-6	Male	Minburi District, Bangkok
45	m-14	KL1	KLMBK 0711-7	Male	Minburi District, Bangkok
46	m-15	KL1	KLMBK 0711-8	Male	Minburi District, Bangkok
47	m-16	KL1	KLMBK 0711-9	Male	Minburi District, Bangkok

Table S1: Details of date palm trees (collected from Horticulture Farm Areas), used for identification and development of sex specific markers (cont.)

No.	Code	Cultivar	Symbol	Sex	Location
48	m-17	KL1	KLMBK 0106-2	Male	Minburi District, Bangkok
49	m-18	KL1	KLMBK 0106-3	Male	Minburi District, Bangkok
50	m-19	KL1	KLMBK 0106-4	Male	Minburi District, Bangkok
51	m-20	KL1	KLMRB 0603-1	Male	Pak Tho District, Ratchaburi
52	m-21	KL1	KLMRB 0603-2	Male	Pak Tho District, Ratchaburi
53	m-22	KL1	KLMRB 0603-3	Male	Pak Tho District, Ratchaburi
54	m-23	KL1	KLMRB 0603-4	Male	Pak Tho District, Ratchaburi
55	m-24	KL1	KLMRB 0603-5	Male	Pak Tho District, Ratchaburi
56	m-25	Barhi	BHMBK 1910-9	Male	Bang Khae District, Bangkok
57	m-26	Barhi	BHMBK 2701-2	Male	Bang Khae District, Bangkok
58	m-27	Barhi	BHMBK 2701-5	Male	Bang Khae District, Bangkok
59	m-28	Barhi	BHMBK 2701-6	Male	Bang Khae District, Bangkok
60	m-29	Barhi	BHMBK 2701-19	Male	Bang Khae District, Bangkok
61	m-30	Barhi	BHMBK 2701-20	Male	Bang Khae District, Bangkok
62	m-31	Barhi	BHMBK 2701-22	Male	Bang Khae District, Bangkok

Table S2: Details of the 45 RAPD primers used for identification of sex specific marker in date palm

No.	Primer Name	Sequences (5´-3´)	No.	Primer Name	Sequences (5´-3´)
1	OPA-12	TCGGCGATAG	24	OPW-18	TTCAGGGCAC
2	OPA-20	GTTGCGATCC	25	OPW-19	CAAAGCGCTC
3	OPB-14	TCCGCTCTGG	26	OPW-20	TGTGGCAGCA
4	OPC-09	CTCACCGTCC	27	OPAA-01	AGACGGCTCC
5	OPD-06	ACCTGAACGG	28	OPAA-02	GAGACCAGAC
6	OPD-10	GGTCTACACC	29	OPAA-03	TTAGCGCCCC
7	OPW-01	CTCAGTGTCC	30	OPAA-04	AGGACTGCTC
8	OPW-02	ACCCCGCCAA	31	OPAA-05	GGCTTTAGCC
9	OPW-03	GTCCGGAGTG	32	OPAA-06	GTGGGTGCCA
10	OPW-04	CAGAAGCGGA	33	OPAA-07	CTACGCTCAC
11	OPW-05	GGCGGATAAG	34	OPAA-08	TCCGCAGTAG
12	OPW-06	AGGCCCGATG	35	OPAA-09	AGATGGGCAG
13	OPW-07	CTGGACGTCA	36	OPAA-10	TGGTCGGGTG
14	OPW-08	GACTGCCTCT	37	OPAA-11	ACCCGACCTG
15	OPW-09	GTGACCGAGT	38	OPAA-12	GGACCTCTTG
16	OPW-10	TCGCATCCCT	39	OPAA-13	GAGCGTCGCT
17	OPW-11	CTGATGCGTG	40	OPAA-14	AACGGGCCAA
18	OPW-12	TGGGCAGAAG	41	OPAA-15	ACGGAAGCCC
19	OPW-13	CACAGCGACA	42	OPAA-16	GGAACCCACA
20	OPW-14	CTGCTGAGCA	43	OPAA-18	TGGTCCAGCC
21	OPW-15	ACACCGGAAC	44	OPAA-19	TGAGGCGTGT
22	OPW-16	CAGCCTACCA	45	OPAA-20	TTGCCTTCGG
23	OPW-17	GTCCTGGGTT			

Supplementary Figures

```
CLUSTAL O(1.2.4) multiple sequence alignment (Aligned. Score: 24.03%)


412      CACTAGATAAGGAAGGGTGTAACCTCATAAGATGGCATATAAGGAAAGAGTATAACAGCT    60
428      -----CCTAAGTAGCTCTCAAACAATAAATC---AGTGGTGACTGGAGAAACAAACTTTT    52
                **** *    *  ***   * *          * *    ***    ***   *

412      TGTTCGAAGATCACAGACTTGATCGTAACAAACTCTCATCCTCCACTCAATATAAACCAG    120
428      GAAGGGAAAGCCTCTTAACTAAGTTTAACAAGTTCTGTCT--CTAACCAGTATGGTTCCT    110
              ***   * *  *  * *   ******  ***      * *  ** ***    *

412      GCTAGGGGA--CCCCAGGTAAAAGAACTTCATCTTGCTCTTAGCTTATTGCTCGTATTAT    178
428      GTAGAGAGTATCAATAAGGAGATTAAAGTCTTTGAG--CTTTGAGTGTTACAACAATA-G    167
         *    * *   *   * * * *  **  ** *   *  *** *  * ** *    **

412      TAGATCTCCATTGTTCCAC-------CAAAACTCTGCTTGACTTAAGCATTAGATGGTGC    231
428      GTTCCCTAAATGGTGCCTACATTAGCTGGGACAGTACACTATGCACTCAATAATCGTTTC    227
              **  ** ** **             **  * *   *   *  ** **   * * *

412      CCTGCTGGACATGTTCGAACAAGGCCTAATTGCTTTTC----TTTGTTTGTA------GG    281
428      CCTAAAAGC--TGCCTATGCAAGAGATAGTTGATTGGGTTTTTTTGGCTGCATTAAGAGA    285
         ***    *   **      ****   ** *** **       ****  ** *      *

412      TTGGATCTTACATCAATATAAATCAAGCCAAGGACCTTCGGTAAAGGAACTTCACCTTGC    341
428      TTGTTCCCCTCATCCTGCATACTCTT-CGAGAGTCCTTAAGTTTCAAAAGTGCTGT---    341
         ***   *   ****      * **   * *  * ****  **     **  *    *

412      TCTTAGCTCATTGCTCGTATTGTTAGATCTC--CATTCTTCCAACAAAACTCTGCT-TGA    398
428      -AGAAAAGCTTGGAGAAAATTATAAAATCATTAGCAGAAGCCACCAAAAAACTGCCTTTT    400
             *   * * *     *** * * ***           *** *****  **** *

412      CTTAAACATTAGAT--------------   412
428      AAGCACTAGTAGATTCCGCCTCCATAAG   428
             *  * *****
```

Supplementary Figure S1. Sequence alignment of the cloned male-specific RAPD fragments of 412 and 428 bp. These two sequences share only 24.03% homology.

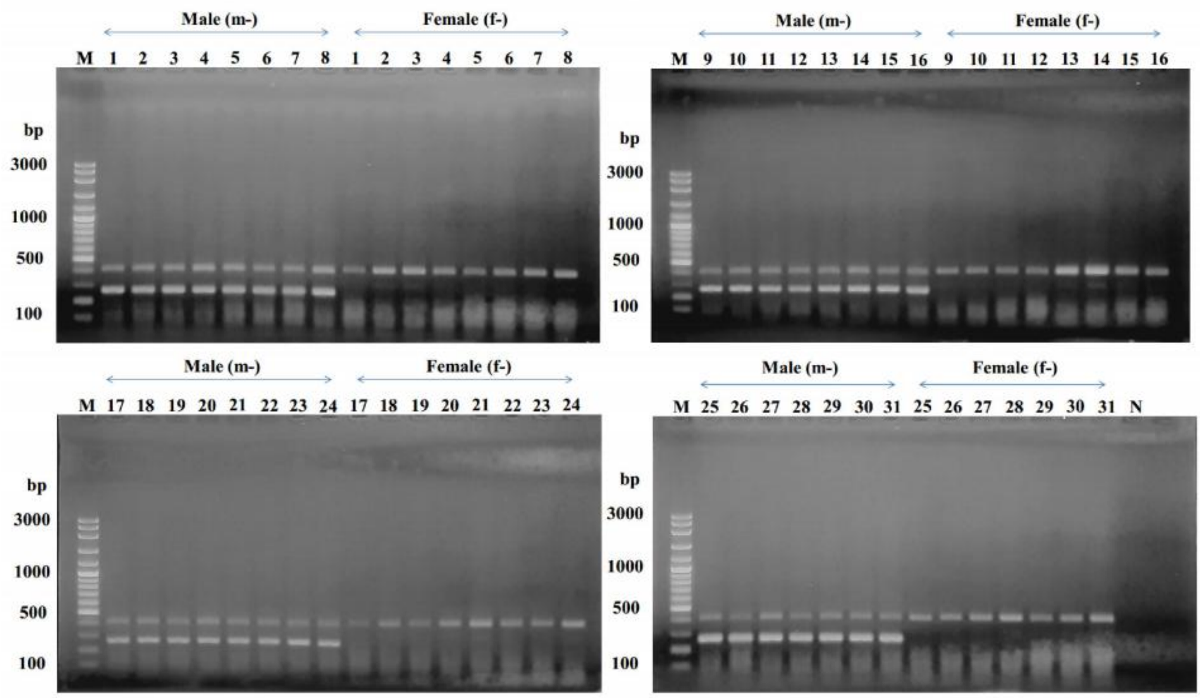

Supplementary Figure S2. Amplicon profiles of male and female date palm individuals of KL1 and Barhi, illustrating the products generated by a multiplex PCR reaction utilizing the mspW18-2F/mspW18-2R primer pair in combination with the mspW18-1F/mspW18-1R primer pair. In the gel lanes, m-1 to m-31 represent male individuals, while f-1 to f-31 female individuals. The letter "M" designates the 100-bp DNA ladder, "N" designates the negative control.
